# Progress and Prospects of Newborn Screening in China

**DOI:** 10.3390/ijns12030072

**Published:** 2026-09-02

**Authors:** Xiaoqiang Hao, Xinwen Huang, Rulai Yang, Xin Yang, Xiaolei Huang, Zhengyan Zhao

**Affiliations:** 1 Department of Endocrinology, Children’s Hospital, Zhejiang University School of Medicine, Hangzhou 310052, China; 2 National Clinical Research Center for Children and Adolescents’ Health and Diseases, Hangzhou 310052, China; 3 Department of Genetics and Metabolism, Children’s Hospital, Zhejiang University School of Medicine, Hangzhou 310052, China

**Keywords:** newborn screening, China, inborn errors of metabolism, next-generation sequencing, tandem mass spectrometry

## Abstract

Newborn screening (NBS) is a critical component of the three-tiered prevention strategy for birth defects, reducing congenital disorder burden and improving long-term child health outcomes. Over the past 45 years, NBS in China has evolved into a nationwide quality-controlled network driven by policy support and technological advances. Currently, phenylketonuria and congenital hypothyroidism are included in universal NBS across the country, and several provinces have expanded to encompass congenital adrenal hyperplasia, glucose-6-phosphate dehydrogenase deficiency, and additional inherited metabolic disorders identified through tandem mass spectrometry. The application of next-generation sequencing and other technologies has further enhanced detection capacity and expanded detectable disease spectra. Meanwhile, the National Quality Management System for NBS (QMS-NBS) has realized the visualization and standardization of screening quality and performance. Despite these advances, challenges remain, including regional disparities, inadequate follow-up, and long-term management. This review summarizes the historical evolution and policy framework of NBS in China, outlines the development of screening institutions, the spectrum and incidence of screened disorders, advances in detection technologies, and the establishment of QMS-NBS. It also highlights future priorities: expanding screened conditions, strengthening follow-up and long-term care, promoting regional equity, and advancing novel technologies to improve child health and foster precision public health.

## 1. Introduction

Newborn screening (NBS), a core component of the three-tiered prevention strategy for birth defects, is widely recognized as one of the most successful initiatives in the global public health field [1,2]. The primary goal of NBS is to detect serious congenital disorders in asymptomatic newborns through sensitive and accurate methods, enabling timely intervention to reduce disability and mortality rates [3,4,5]. It is estimated that approximately 140 million newborns worldwide undergo NBS annually, with over 30,000 of these infants gain significant clinical benefits from this critical measure [6,7]. Broadly defined, NBS also encompasses hearing screening and screening for critical congenital heart disease; in this review, however, NBS specifically refers to blood spot-based screening for inborn errors of metabolism (IEMs).

Global NBS originated in the 1960s, initially targeting a limited number of treatable IEMs such as phenylketonuria (PKU) and congenital hypothyroidism (CH). Using dried blood spot (DBS) samples, early programs covered a relatively narrow range of disorders [8]. A breakthrough in NBS was achieved with the application of tandem mass spectrometry (MS/MS) technology, which enabled simultaneous detection of multiple conditions from a single DBS sample and substantially expanded the NBS spectrum [9,10,11]. More recently, rapid advances in next-generation sequencing (NGS) have further extended the boundaries of NBS. Multiple countries and regions are now exploring the integration of whole-exome sequencing and whole-genome sequencing (WGS) to achieve broader and more precise early detection of genetic diseases [12,13].

In China, NBS began in the early 1980s with pilot programs in selected cities. International collaboration and domestic policy support facilitated rapid expansion of coverage, eventually achieving nationwide implementation. Concurrently, national NBS laboratories were integrated into a unified quality management framework, achieving standardized testing through proficiency testing and external quality assessment [14]. Guided by legal and administrative regulations, China has established a three-tiered screening network that covers the entire country, forming a closed-loop process encompassing sample collection, laboratory testing, diagnostic recall, treatment, and follow-up management. At present, the annual screening volume has exceeded 10 million cases, covering eastern, central, and western regions, making it one of the largest and most comprehensive NBS systems worldwide.

This review provides a systematic overview of China’s NBS infrastructure and operational mechanisms, summarizes its current status and developmental characteristics, and provides an evidence base for optimizing screening strategies and strengthening the birth defect prevention and control system. By contextualizing China’s experience within the global development of NBS, it also aims to inform the refinement of screened conditions, promote the standardized application of genomic technologies, and offer a scalable “China model” for birth defect prevention and child health worldwide.

## 2. Historical Trajectory and Policy Evolution of NBS in China

### 2.1. Early Development and Expansion of NBS in China

China’s NBS originated in 1981 with pilot screening for CH, PKU, and galactosemia at Shanghai Xinhua Hospital [1]. Subsequently, a multi-center study led by the First Hospital of the former Beijing Medical University (now Peking University Health Science Center) screened approximately 200,000 newborns for PKU across 11 regions, providing the first multi-regional PKU epidemiological baseline (incidence ~1:16,500), laying the scientific foundation for national rollout.

### 2.2. International Collaboration and Establishment of National Screening Capacity

In the late 1990s, international collaborations accelerated the transition from pilot programs to organized screening systems. Beginning in 1996, China launched a two-phase collaboration with the Ministry of Social Affairs and Health of Finland. The first three-year phase established screening laboratories, provided essential equipment, delivered professional training, and developed regional screening networks across Shanghai, Jiangxi, Henan, and Tianjin, with a focus on CH and PKU. By 2004, the five participating centers had screened approximately 1.3 million newborns, identifying 512 CH cases and 80 PKU cases. A second five-year phase (2006–2011) extended to seven additional provinces, aiming to raise the hospital-based NBS rate above 50% by 2010-a target largely achieved by the program’s completion [15].

In parallel, the International Atomic Energy Agency (IAEA) launched the “Regional Screening Network for Neonatal Hypothyroidism” (Project RAS/6/032) in 1999, with Tianjin serving as a key participating center [16]. In the same year, China joined the first regional NBS meeting of Asian countries in Korea, as one of 11 East Asian member states [17,18]. Through technical cooperation, the IAEA facilitated the standardization of CH screening and workforce development via professional training fellowships and translated technical guidelines. Even after IAEA funding concluded in 2008, China continued to engage in regional NBS exchanges, sharing national strategies within the network.

Together, these international partnerships, combined with domestic policy efforts, drove the national NBS rate from approximately 20% to 50% [1], while establishing the foundational sample-to-follow-up workflow and diversifying screening methods from limited and inefficient to standardized and efficient.

### 2.3. Development of Legal and Regulatory Frameworks

**Figure 1:**
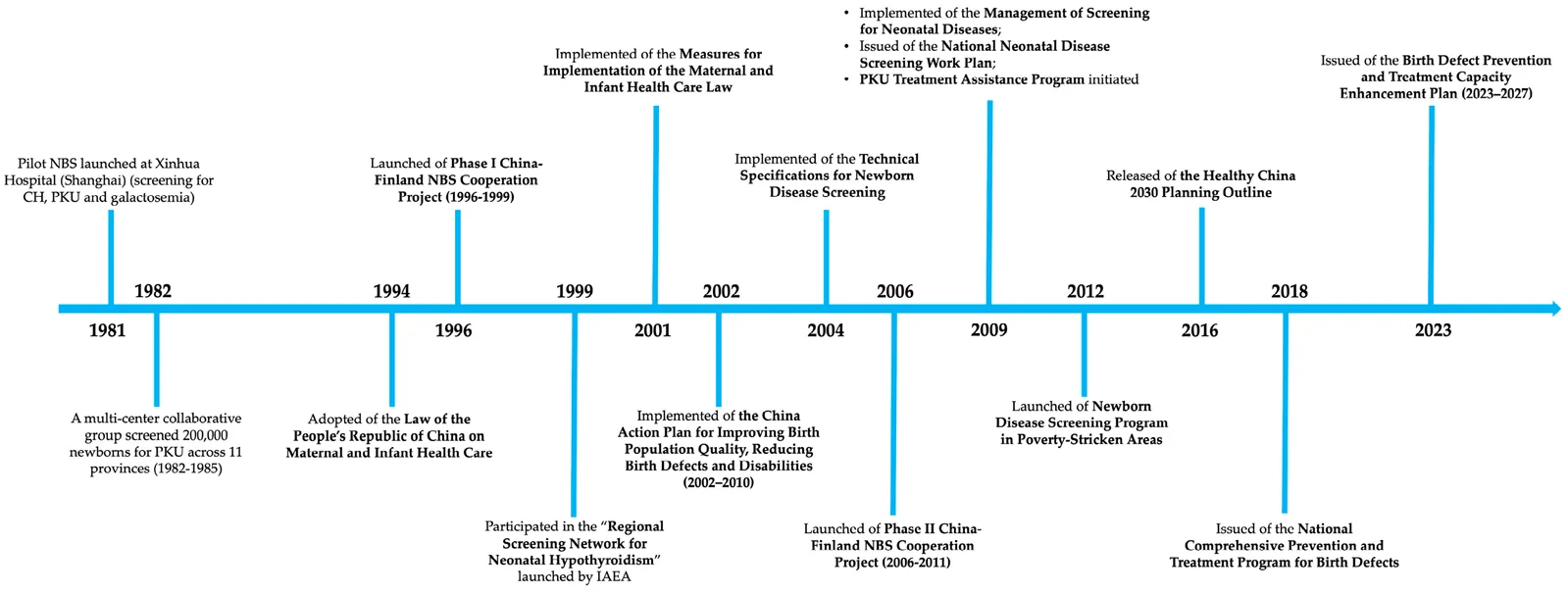
Historical timeline and policy milestones of China’s NBS development (1981–2023). The timeline highlights key clinical pilots, international technical cooperation projects, legislative enactments, national work plans, and regional assistance programs that drove the evolution of China’s NBS system over the past four decades. Abbreviations: CH, congenital hypothyroidism; NBS, newborn screening; IAEA, International Atomic Energy Agency; PKU, phenylketonuria.

Since the 1990s, the Chinese government has promoted the systematic construction of a legal guarantee system for NBS through top-level design. Continuous improvements in legislation and institutional innovation have facilitated the standardization of NBS nationwide, as detailed in Table 1 and visualized in Figure 1.

### 2.4. Regional Implementation and Local Policy Innovation

Building upon national regulations, provincial and municipal governments have developed complementary policies according to local healthcare resources, disease spectra, and administrative capacity. These regional approaches have contributed to the diversification and optimization of NBS implementation. Local policy measures can be broadly classified into three categories:

#### 2.4.1. Local Regulations

Many provinces and cities have incorporated NBS into their maternal and infant health legal frameworks, establishing a provincial-level legal guarantee system. Notable examples include the “Beijing Municipal Implementation Measures for the Law of the People’s Republic of China on Maternal and Infant Health Care” [34], the “Shanghai Municipal Maternal and Infant Health Care Regulations” [35], and the “Sichuan Provincial Law of the People’s Republic of China on Maternal and Infant Health Care” [36].

#### 2.4.2. Local Administrative Measures

Building on national standards, provinces like Zhejiang, Jiangsu, Shanghai, Shandong, Hebei, Hunan, Sichuan, Gansu, and other regions have refined local NBS implementation regarding procedures, quality control, and management. Notably, Guangdong expanded mandatory screening to include glucose-6-phosphate dehydrogenase (G6PD) deficiency, congenital adrenal hyperplasia (CAH), congenital heart disease, and retinopathy of prematurity beyond national mandates, while establishing a tiered network with strengthened supervision [37]. These local policy innovations not only broaden the range of screened conditions but also promote the standardized and high-quality development of regional screening systems.

#### 2.4.3. Periodic Work Plans or Implementation Plans

Some provinces periodically issue phased Work Plans or Implementation Plans to dynamically adjust targets and tasks, optimize resource allocation, and improve screening services. Zhejiang Province, for example, expanded free screening for inherited metabolic disorders from four conditions to 29 conditions while integrating hearing and congenital heart disease screening into a broader newborn health package [38].

Overall, China’s NBS governance has evolved into a “national guidance–provincial implementation–dynamic adjustment” model, enabling the effective translation of macro-level policy into scalable public health practice.

## 3. Infrastructure and NBS System Development

As an interdisciplinary public health endeavor, the effective operation of NBS depends on the coordinated functioning of a multi-tiered organizational network, standardized technical workflow, and comprehensive quality control across all stages.

### 3.1. Organizational Management System: A Three-Tier Collaborative Governance Model

China’s NBS program operates under a three-tiered collaborative management system encompassing the national, provincial, and prefectural/county levels. Table 2 details the specific responsibilities at each level.

**Figure 2:**
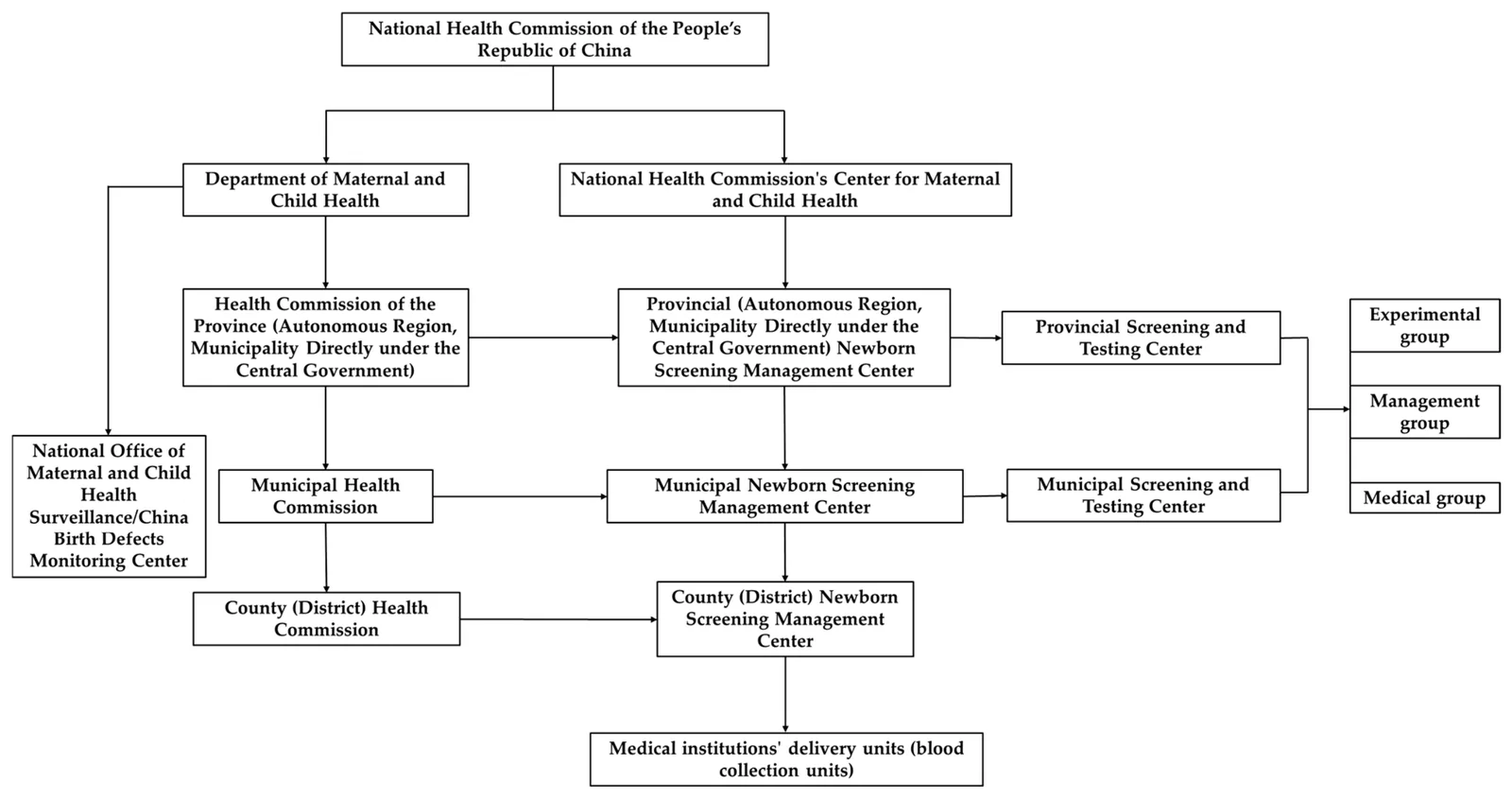
Organizational structure of NBS program in China.

Based on this governance structure, China has established a nationwide network of 269 NBS centers (Figure 2) [1]. Provincial organizational models vary from multi-center systems to centralized operations. For instance, Zhejiang Province employs a centralized model through the Children’s Hospital Zhejiang University School of Medicine, which processes more than 700,000 NBS annually.

### 3.2. Institutional Setup and Screening Pathway: Full-Process Management

**Figure 3:**
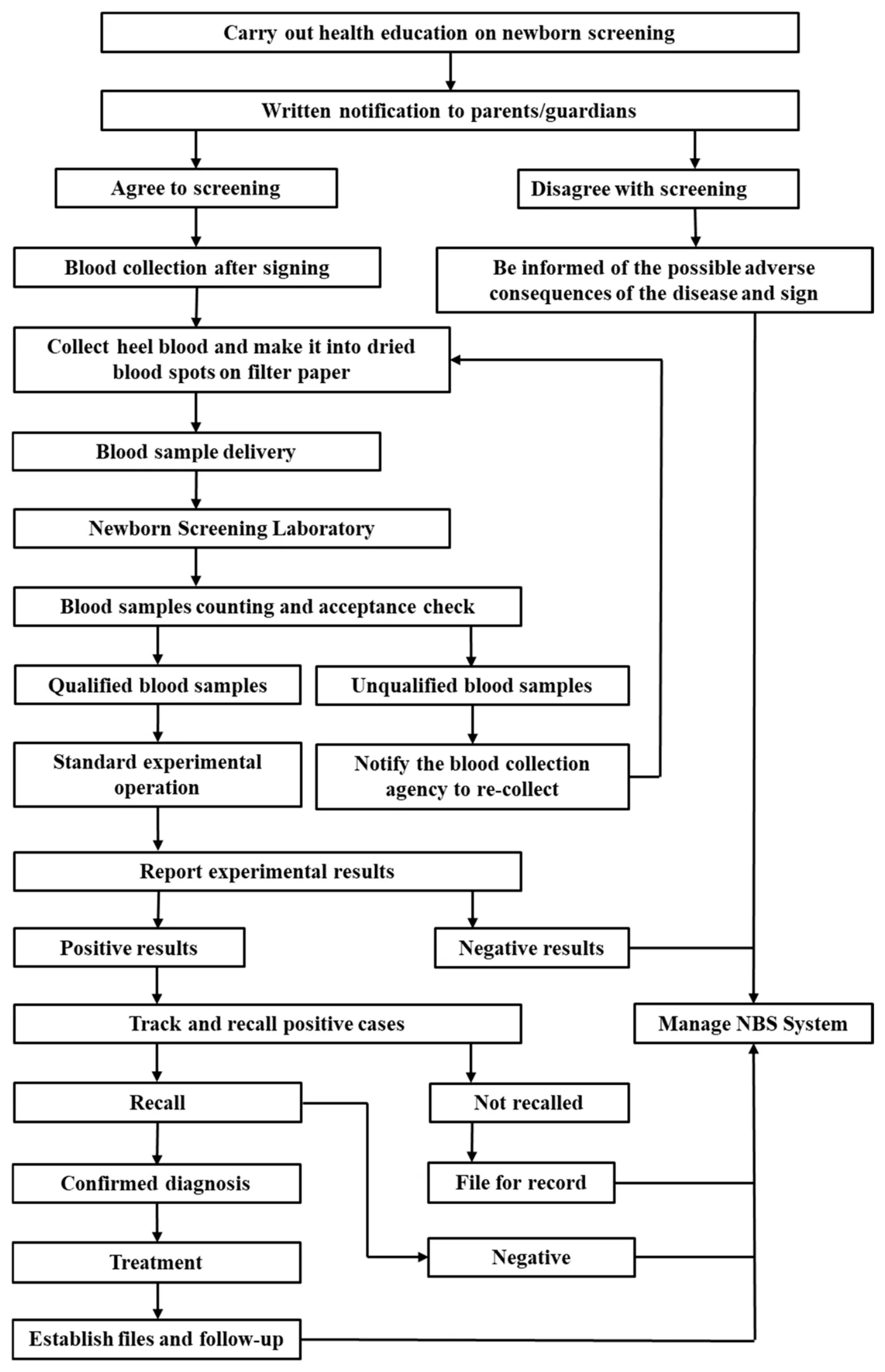
Workflow of the NBS program in China.

China’s NBS system has developed a standardized workflow encompassing screening, diagnosis, and intervention, characterized by timeliness and traceability, with clearly defined responsibilities across three categories of institutions (Figure 3).

Blood Collection Institutions (medical institutions with obstetrics/pediatrics departments): Responsible for health education, obtaining informed consent, heel blood sample collection, quality self-checks, and standardized sample delivery. Substandard or missing samples must be promptly re-collected; records retained for ≥10 years.

Screening Laboratories (provincial-designated, certified centers with annual capacity ≥ 30,000 cases [39]): Responsible for sample registration within 24 h, standardized testing and interpretation, report issuance, and adherence to internal/external quality control requirements.

Diagnosis and Treatment Institutions (provincial-designated clinical centers): Responsible for confirmation, treatment, and follow-up of screen-positive infants, integrating confirmed cases into the 0–6-year high-risk child health management system. Records retained for ≥10 years.

This screening pathway adopts a closed-loop management model that spans health education, informed consent, sample collection and transportation, laboratory testing, result reporting, recall, diagnosis, intervention, and follow-up. It highlights timeliness, standardization, and traceability to ensure that affected infants receive timely and effective interventions.

### 3.3. Quality Control and Management of Screening Laboratories: A Multi-Level Technical Safeguard

Since NBS relies on identifying affected infants through laboratory testing before clinical symptom onset, rigorous laboratory quality management is paramount. Laboratories must comply with the “Administrative Measures for Clinical Laboratories in Medical Institutions” [40] and “Medical Laboratories-Requirements for Quality and Competence” [41], implementing quality assurance through Internal Quality Control (IQC), External Quality Assessment/Proficiency Testing (EQA/PT), and screening data comparison [42,43].

#### 3.3.1. IQC

Precision and accuracy are monitored via quality control charts, with regular instrument calibration, reagent verification, and monitoring of key indicators (e.g., unqualified DBS rate, recall rate, detection rate). Deviations trigger immediate retesting; records retained for ≥10 years.

#### 3.3.2. EQA/PT

EQA/PT follows ISO/IEC 17043 [44] and CNAS CL03 standards [45]; laboratories are encouraged to obtain ISO 15189 certification [46]. The National Expert Group on Neonatal Screening has established 16 core quality indicators, with regular reporting to the National Center for Clinical Laboratories (NCCL) [47,48].

Since 1998, NCCL has organized proficiency testing for screening centers nationwide, with eight National Summation Conferences held in 2005, 2007, 2009, 2011, 2013, 2017, 2019, and 2023. Table 3 summarizes the substantial growth in laboratory participation across major testing categories [49]:

To systematically manage laboratory quality, NCCL has established the Quality Management System for the NBS Network, comprising two components [49]:

1. Quality Indicator System for the Entire NBS System (QIS-NBS)

For NBS institutions, the QIS-NBS comprises three first-level indicators, 15 second-level indicators, and 68 third-level indicators, covering the entire process of quality management such as institutional setup, personnel, laboratory infrastructure, information systems, pre-screening publicity and education, sample collection quality, laboratory testing, post-test management, sample preservation, case management, follow-up, and treatment effectiveness.

For blood collection institutions, the system includes three first-level indicators, seven second-level indicators, and 16 third-level indicators, mainly assessing institutional and personnel development, health education and outreach, sample collection, informed consent, sample quality, and material preservation.

2. Quality management implementation program for the NBS Network (QMIP-NBS)

The QMIP-NBS is a structured work procedure incorporating a range of tools to guide and standardize the control and assessment work of administrative bodies and service institutions [49].

### 3.4. Information Management System: An Efficiently Operating “Data Infrastructure”

NBS’s multi-disciplinary, multi-step nature demands robust information management:

#### 3.4.1. Screening Information System Architecture and Core Modules

The system adopts a Browser/Server architecture, with the entire process management as the core; the system integrates the modules of screening workflow, quality control, logistics, diagnosis and treatment, and finance. This enables efficient data flow across the provincial, prefectural, and county levels.

#### 3.4.2. Core Business Processes

The system comprehensively controls quality across the pre-analytical, analytical, and post-analytical phases, implementing information-based tracking for all steps from blood collection, transportation, and testing to diagnosis, treatment, and follow-up. Confirmed cases are incorporated into electronic records and trigger follow-up reminders, while negative results are classified into routine management.

#### 3.4.3. Systems Operation, Maintenance, and Security Management:

A comprehensive operational and maintenance framework, combined with a five-tier security protection mechanism, safeguards the system across multiple dimensions such as physical, network, host, application, and data security, ensuring data privacy and stable system performance.

### 3.5. Professional Collaboration and Technical Support: Professional Leadership and Sustained Development

The Chinese Society of Neonatal Screening and its expert group drive academic and technological advances in NBS. Their core responsibilities include formulating national technical standards and clinical guidelines, organizing national conferences and professional training, and hosting annual quality assessment workshops to foster regional knowledge exchange. Furthermore, the Society conducts regional symposia, offers online education, provides remote technical guidance, and supports NBS implementation in underserved regions to promote equity.

Additionally, three authoritative monographs led by Professor Zhengyan Zhao (“Newborn Disease Screening [15],” “Newborn Genetic Metabolic Disease Screening [50],” and “Newborn Genetic Screening [51]“) serve as essential references in China. By introducing advanced international technologies, these societies continuously advance China’s NBS system toward standardization, professionalization, and high-quality development.

## 4. Current Status of NBS in China

With the continuous enhancement of infrastructure and screening systems, and the integration of advanced technologies such as MS/MS and NGS, the spectrum of diseases included in NBS in China has expanded substantially.

### 4.1. Core Conditions

**Figure 4:**
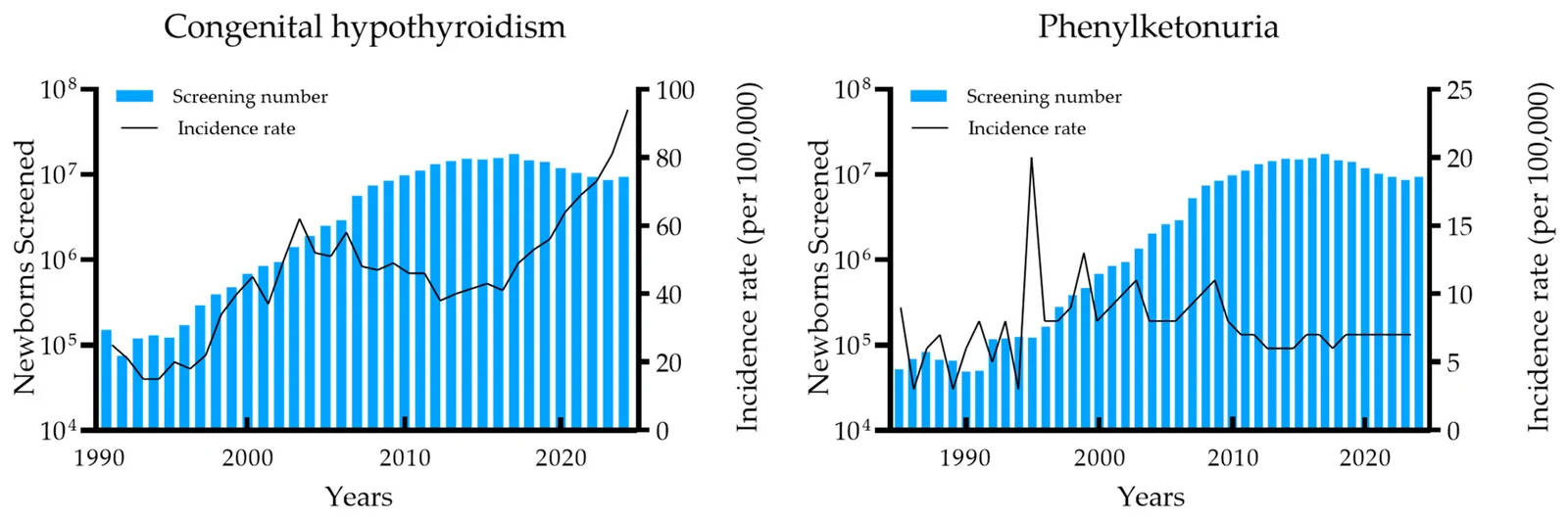
Newborns screened and incidence rates of CH and PKU in the NBS program in China, 1985–2024.

China has incorporated PKU and CH into the national mandatory screening panel, making them the core of the NBS program. Currently, all 31 provinces (autonomous regions and municipalities directly under the central government) conduct PKU and CH screening. The screening rate has increased from 69.8% in 2011 to 97.9% in 2022, with rates reaching 99.4% in eastern China, 98.5% in central China, and approximately 95.6% in western China [1]. The screening situations of PKU and CH in previous years are shown in Table 4 and Figure 4.

PKU: Research from the National Office for Maternal and Child Health Surveillance indicates that Gansu Province has the highest PKU incidence rate (0.29‰), followed by Ningxia Hui autonomous region (0.19‰) and Qinghai province (0.17‰), while the lowest incidences were observed in Guangdong province (0.0053‰) and Guangxi Zhuang autonomous region (0.0015‰) [52]. Global Moran’s I analysis indicates a significant spatial autocorrelation of PKU cases [52]. Local Moran’s I analysis reveals that high-high clustering areas are concentrated in Gansu, Ningxia, Qinghai, and Shanxi provinces, whereas seven southern provinces (Guizhou, Hunan, Jiangxi, Guangxi, Guangdong, Fujian, and Hainan) form low-low clustering areas. These findings further emphasize the need for more screening and early diagnosis measures in northern China, as well as additional supportive interventions for families of affected children [52].

CH: A retrospective study covering 92 million newborns from 2013 to 2018 shows that CH incidence exhibits moderate positive global spatial autocorrelation (Global Moran’s I = 0.394, *p* < 0.05) with significant spatial clustering. The CH clustering areas include 11 provinces/municipalities: Anhui, Fujian, Guangdong, Henan, Hubei, Hunan, Jiangxi, Jiangsu, Shandong, Shanghai, and Zhejiang [53]. An analysis of data on 110 million newborns by the Chinese Birth Defects Monitoring Center reveals that central China has the highest average annual growth rate of CH (6.54%), followed by western China (5.46%) and eastern China (4.75%) [54].

Beyond the core conditions, China has significantly expanded its screening capacity. As of 2022, 23 provinces have implemented screening for G6PD deficiency (screening rate: 89.24%), and 24 provinces have conducted screening for CAH (screening rate: 91.45%) [1].

G6PD deficiency: Data from 2013 to 2017 show that the overall incidence of G6PD deficiency ranges from 0.67% to 0.77%, following a clear south–north gradient with higher rates in southern regions. Guangxi Zhuang Autonomous Region has the highest incidence (3.387%), while Shanxi Province has the lowest (0.008%). Southern provinces (such as Guangdong, Hainan, and Jiangxi) generally have higher incidence rates than northern provinces [55].

CAH: Based on data from 7.85 million newborns, the overall incidence of CAH is 0.43‱ (approximately 1 in 23,024). The incidence rates in Zhejiang, Guangdong, Hubei, and Shaanxi provinces are lower than the national average, while rates in other regions are higher than the average [56].

### 4.2. Expanded Screening for IEMs

In the mid-1990s, MS/MS was applied to NBS for IEMs, significantly expanding the range of screened diseases. Since the establishment of China’s first MS/MS-based IEM screening laboratory in 2002, by 2025, a total of 356 laboratories across 29 provinces (autonomous regions and municipalities directly under the central government) nationwide have adopted this technology for screening [1]. For instance, in Zhejiang Province, the “Zhejiang Province Newborn Disease Screening Project Implementation Plan (2023 Edition)” expanded the scope of IEM screening from 4 to 29 diseases, covering CH, CAH, G6PD deficiency, 9 amino acid metabolism disorders, 8 fatty acid oxidation disorders, and 9 organic acid metabolism disorders [38].

#### 4.2.1. Incidence of IEMs

An early pilot study in 2004 estimated the overall incidence of IEMs to be 1 in 3795 live births [57]. A 2020 national survey, based on 2016–2017 data from 246 NBS centers and conducted by the National Birth Defects Monitoring Center, reported an overall IEM incidence of 1 in 2585 live births [58]. Among IEMs, amino acid disorders were the most prevalent, with an incidence of 17.14 per 100,000 births (95% CI: 16.21–18.13), followed by organic acid disorders (12.39 per 100,000 births, 95% CI: 11.60–13.24) and fatty acid oxidation disorders (9.16 per 100,000 births, 95% CI: 8.48–9.89) [58].

#### 4.2.2. Regional Differences in IEM Incidence

There were significant regional differences in the overall incidence of IEMs and disease-specific incidence (*p* < 0.001). The cumulative incidence was highest in northwestern China, followed by southwestern China and southern China [58]. Beyond this national-level regional analysis, city- and province-specific screening data further illustrate the extent of this variation, with reported IEM incidence ranging from as high as 1 in 1476 (Xinjiang) to as low as 1 in 4503 (Zhejiang) across different cohorts (Table 5).

Region-specific incidences of individual IEMs also differ: hyperphenylalaninemia was the most prevalent amino acid disorder, with the highest incidence in northwestern China; methylmalonic acidemia is most common in southwestern China; and primary carnitine deficiency predominates in southern China.

These patterns may be associated with ethnic genetic backgrounds, MS/MS screening coverage, recall rates of suspected positive cases, and variations in screening panels [58]. Critically, most newborns screened by MS/MS were concentrated in eastern China, with only a small proportion screened in northeastern and southwestern China—a coverage imbalance that likely contributes to, rather than merely correlates with, the observed regional incidence differences. Consistent with this caveat, northeastern China, where screening coverage is lowest, also showed the lowest cumulative and disease-specific incidence rates [58], raising the possibility that this apparent “low burden” partly reflects under-ascertainment rather than true low prevalence.

The observed regional disparities imply that the cost-effectiveness and feasibility of expanded screening programs may vary geographically. A uniform nationwide expansion may not be optimal. Instead, continuous collection of local epidemiological data is essential to inform tailored screening strategies, prioritizing diseases with high local prevalence and optimizing resource allocation.

### 4.3. Emerging Screened Conditions

In recent years, several provinces in China have piloted the inclusion of more diseases that can be screened and intervened in NBS, such as lysosomal storage diseases (LSDs) [69,70,71], Duchenne muscular dystrophy (DMD) [72,73,74,75], spinal muscular atrophy (SMA) [76], severe combined immunodeficiency (SCID) [76], X-linked Agammaglobulinemia (XLA) [76], and X-linked adrenoleukodystrophy (X-ALD) [77].

LSDs: In China, several studies based on MS/MS have reported relatively high detection rates of LSDs. A screening study of 50,108 newborns in Shanghai showed an overall incidence of 1 in 1856 [71], while Nanjing Drum Tower Hospital reported a detection rate of 1 in 1512 among 22,687 newborns [69]. In contrast, relevant studies in Taiwan (64,148 newborns, 1 in 4277) [78] and Italy (112,446 newborns, 1 in 4498) [79] reported lower LSD incidences than those observed in these Chinese reports.

SCID/XLA/SMA: Regarding primary immunodeficiencies and neuromuscular diseases, a comprehensive screening study using multiplex real-time PCR technology conducted by the Children’s Hospital Zhejiang University School of Medicine simultaneously evaluated the incidence of SCID, XLA, and SMA. The results showed that the estimated prevalence of SCID was 1 in 103,240, XLA was 1 in 51,620, and SMA had a relatively higher estimated prevalence of 1 in 11,471 [76].

DMD: Regional pilot studies indicate variability in DMD incidence of DMD among male newborns. The estimated incidence rates in Ningxia Hui autonomous region and Henan province are similar, at 1 in 5000 [74] and 1 in 4370 [72] respectively, whereas Guangdong province reports a lower incidence of 1 in 11,067 [73]. These findings, derived mainly from local studies, require validation through larger population-based cohorts.

X-ALD: In a screening study of 43,653 newborns in Guangdong province, the incidence among males was approximately 1 in 3324 [77], markedly higher than reported in the United States (1 in 10,500) [80] and Taiwan (1 in 13,825) [81], suggesting potential population-specific epidemiological differences.

### 4.4. Integration of NGS

In recent years, NGS technology has been progressively applied in NBS. It is particularly suitable for diseases lacking biochemical markers, enabling early detection, rapid diagnosis, and carrier screening, while also providing reproductive guidance for families. Domestically, multiple pilot studies have explored the combined application of NGS and MS/MS to enhance detection efficiency and diagnostic accuracy [82,83,84].

In 2021, the NBS project launched by the Children’s Hospital Zhejiang University School of Medicine targeted 135 genes associated with 75 congenital diseases. A large-scale, multi-center, prospective multi-disease genetic testing analysis was conducted on DBS samples from 21,442 newborns across 12 hospitals in 6 provinces (autonomous regions and municipalities directly under the central government) nationwide. Pathogenic genetic variants were detected in 5700 newborns, among whom 168 cases (0.78%) were phenotypically positive [85]. The Beijing Children’s Hospital Affiliated to Capital Medical University conducted a retrospective screening using targeted sequencing on 11,484 newborns; the cohort achieved a clinical diagnosis rate of 0.95%, with the preliminary reported turnaround time (including a sequencing cycle of < 7 days) completed within 11 days [86]. A multi-center study led by Xinhua Hospital Affiliated to Shanghai Jiao Tong University School of Medicine implemented combined genetic and traditional biochemical screening for 29,601 newborns, achieving a positive predictive value (PPV) of 50.4% (95% CI: 50.0–53.9%) [84]. This suggests that genetic testing can enhance the detection capability of traditional screening in the general newborn population. A study by Nanjing Maternity and Child Health Care Hospital compared the screening effectiveness of traditional NBS and genetic screening in healthy newborns and high-risk infants. The results showed that genetic screening significantly reduced the false-positive rate in high-risk infants (0.22% vs. 3.47%) and improved the PPV (60.0% vs. 5.88%) [87].

In summary, findings from these multi-center studies provide important references for the disease spectra in different regions of China. They indicate that genetic screening not only serves as a valuable supplement to traditional screening but also demonstrates greater clinical benefits in high-risk populations. Internationally, exploratory newborn genomic screening programs have also been launched in the United States and Europe, such as the BeginNGS project in the US [88,89], the Genomic Uniform-screening Against Rare Disease in All Newborns (GUARDIAN) project [90], and the BabyDetect study in Belgium [91]. These studies consistently show that genetic testing can identify diseases not covered by traditional biochemical screening and provide additional value in carrier detection and family-level guidance. However, international experience also reveals challenges associated with genetic screening in areas such as ethics, result interpretation, cost-effectiveness, and information feedback. Therefore, while China’s pilot data provide valuable proof-of-concept evidence, broader NGS adoption in NBS should proceed cautiously, informed by region-specific disease spectra, economic capacity, and—critically—by prospectively designed studies that report on the ethical and cost-effectiveness dimensions largely absent from current Chinese literature.

## 5. Guidelines, Consensus Statements, and Diagnostic-Therapeutic Standards

With the continuous expansion of diseases covered by NBS in China, screening has gradually extended from traditional core disorders to a broader range of IEMs and other congenital conditions. This expansion has correspondingly increased the complexity and technical demands of the screening process. To address these challenges, numerous technical guidelines and expert consensuses, led by national authorities or professional organizations, have been issued in recent years, promoting the standardization and regularization of China’s NBS system.

### 5.1. Single Condition Protocols

To address the screening disease-specific screening needs, a series of targeted guidelines and consensuses have been progressively developed, providing a basis for the precision of single-disease screening and clinical management:

- Clinical Practice Guidelines for Phenylketonuria (2020) [92];
- Chinese Expert Consensus on the Diagnosis and Treatment of Fabry Disease (2021 Edition) [93];
- Expert Consensus on Newborn Screening for Spinal Muscular Atrophy (2023) [94];
- Guidelines for the Diagnosis and Treatment of Congenital Hypothyroidism (2025) [95].

### 5.2. Technical Standards

To ensure screening quality, a standardized system covering sample processing, detection methods, and the application of new technologies has been established:

- Expert Consensus on the Management of Biological Samples for Newborn Disease Screening (2020) [96];
- Chinese Consensus for Newborn Genetic Screening of Next Generation Sequencing in the Screening of Monogenic Diseases (2023) [97];
- Consensus on the Organizational Management and Dry Blood Sample Collection for Neonatal Inherited Metabolic Disease Screening [39];
- Consensus on Laboratory Testing Technical Guidelines of Neonatal Genetic Metabolic Disease Screening [98];
- Expert consensus on the combined screening of genes and biomarkers for neonatal diseases (2025) [99].

### 5.3. Follow-Up and Life-Cycle Management

Follow-up and management after screening are key links to connect the “early screening-early intervention” chain:

- Expert Consensus on the Follow-up of Newborn Screening for Neonatal Genetic and Metabolic Diseases (2020) [100].
- Consensus on Diagnosis and Treatment of Inherited Metabolic Diseases by Newborn Screening (2023) [101].

The release of these guidelines, consensuses, and standards has promoted the precision of single-disease screening, the standardization of technical applications, and the regularization of whole-cycle screening management. They provide important technical support for the high-quality development of China’s NBS system and lay the foundation for further reducing the incidence of birth defects.

## 6. Screening Costs and Payment Mechanisms

After over four decades of development, China’s NBS has formed a multi-level service system guided by national policies and implemented independently by local governments. However, due to China’s vast territory, uneven economic development, and disparities in medical resource distribution, significant differences remain across regions in terms of the scope of screened diseases, cost standards, and payment methods.

### 6.1. Regional Differences in Screening Costs

NBS costs in China vary significantly depending on the type of program and region:

Basic screening programs: These mainly include PKU and CH, with some regions also including G6PD deficiency and CAH. The cost is relatively low, approximately $7.43 to $18.52 [57].

Expanded MS/MS screening: The cost of routine MS/MS screening is $50 to $100 per infant [57]. Provinces and cities set standards based on their economic conditions, fiscal status, and screening program scope. For example, in accordance with the “Zhejiang province newborn disease screening project implementation plan (2023 Edition)”, Zhejiang province uniformly adjusted the NBS fee standard to CN¥328 per infant, including CN¥268 for IEM screening, CN¥45 for hearing loss screening, and CN¥15 for congenital heart disease screening [38].

Newborn genetic screening (NBGS): In addition to standard screening programs, the market for NBGS is growing, with generally higher prices—families usually need to bear CN¥500 to over CN¥2000 out of pocket. Surveys indicate that most families are willing to pay approximately CN¥1000 to CN¥2000 for expanded genomic screening [102].

### 6.2. Regional Differences in Screening Payment Methods

Payment methods for NBS in China vary significantly across regions, mainly including government finance-led payment, limited involvement of medical insurance, supplementation by public welfare and social forces, and individual out-of-pocket payments.

Government finance-led payment: Most provinces integrate the core screening programs in their “Basic Public Health Service Programs”; in some regions, screening costs are fully covered by public finance (e.g., Kunshan in Jiangsu province, Mianyang and Chengdu in Sichuan province) [103,104,105]. Funding sources typically adopt a multi-level fiscal sharing mechanism. For example, in some areas of Hunan province, the cost is shared by provincial, municipal, and district finances at a ratio of 6:2:2 [106]; while in Jiangxi province, the cost is co-funded by provincial and municipal/county finances at a ratio of 3:7 [107].

Limited involvement of medical insurance and maternity insurance: The diagnosis and treatment of all genetic metabolic diseases are eligible for medical insurance reimbursement. For instance, the Urban Resident Basic Medical Insurance in Fuzhou and Hefei can reimburse 70% or more of PKU treatment costs [108]. However, this falls under guarantee for the treatment of specific diseases, not payment for universal screening costs. In July 2025, the National Healthcare Security Administration issued three major welfare policies for newborns’ medical insurance enrollment, focusing primarily on the timeliness of newborns’ insurance registration and access to inpatient benefits; these policies also did not directly address the reimbursement of screening costs [109].

Public welfare foundations and social participation: Beyond the government-led system, public welfare foundations and social organizations play an important role in supplementing the NBS payment system. For example, the “China Birth Defect Intervention and Assistance Foundation”, relying on public funds such as central special lottery public welfare funds, implements large-scale screening, diagnosis, and assistance programs nationwide, effectively compensating for gaps in government public health service coverage. Its projects include the “Newborn Multiple Inborn Metabolic Disease Testing Project,” “Congenital Structural Deformity Assistance Project,” “Inborn Metabolic Disease Assistance Project,” and “Thalassemia Assistance Project” [110]. This model, through centralized and professional management of social donation resources, significantly enhances the credibility, coverage, and sustainability of assistance projects, providing an official, transparent, and efficient channel for social participation.

Enterprise participation: Enterprises support screening initiatives through various forms such as direct donations, establishing special funds, or corporate social responsibility projects, promoting resource innovation and model diversification in this field.

Social groups and individual assistance: The main function of social groups and private individual fundraising is to provide emergency financial assistance to families with diagnosed infants, rather than supporting the regular operation of the screening system.

Individual out-of-pocket payments: Currently, individual out-of-pocket payments mainly consist of small fees charged for basic screening and self-funded expenses incurred when families voluntarily choose expanded screening projects beyond the scope of government funding.

## 7. Challenges and Future Perspectives

Despite substantial progress in screening coverage, infrastructure development, and technological advancement, China’s NBS system continues to face challenges related to equitable access, evidence-based expansion, technological integration, and long-term management. Future development should move beyond coverage-oriented expansion toward a more sustainable, evidence-based, and patient-centered model that integrates screening, diagnosis, treatment, and lifelong follow-up.

### 7.1. Equity of Access: Regional, Institutional, and Economic Disparities

Although China has established a nationwide NBS framework, regional disparities in laboratory capacity, healthcare resources, epidemiological data, and financing mechanisms continue to affect equitable access. Resource-limited regions often face shortages of trained personnel, diagnostic capacity, and high-quality surveillance data, which may further hinder evidence-based resource allocation.

Financial accessibility is another important challenge. Current payment mechanisms vary substantially across regions, and expanded MS/MS or NBGS programs are not uniformly covered. The increasing availability of self-funded NBGS may introduce additional socioeconomic disparities if access depends largely on family financial capacity.

Future priorities should include strengthening national surveillance and data-sharing platforms, improving sustainable financing mechanisms for screening services, and optimizing regional laboratory networks to enhance both efficiency and accessibility.

### 7.2. Evidence-Based Screening Expansion and Technological Standardization

The expansion of screening panels has increased the potential of NBS but also introduced challenges in disease selection, clinical management, and quality assurance. Currently, differences in screening panels among provinces reflect local innovation but also highlight the need for a unified, evidence-based evaluation framework incorporating disease prevalence, screening performance, clinical utility, available interventions, and cost-effectiveness. In some cases, screening expansion has progressed faster than the development of corresponding diagnostic and therapeutic guidelines, potentially limiting the clinical benefit of early detection. Therefore, the inclusion of new conditions should be accompanied by standardized clinical pathways and follow-up strategies.

For NGS-based NBS, although genomic approaches may expand disease detection and improve etiological diagnosis, their broader implementation requires evidence beyond diagnostic yield. Current Chinese pilot studies have mainly focused on detection performance, whereas cost-effectiveness, secondary-finding disclosure strategies, psychological impact on families, and long-term clinical outcomes remain insufficiently assessed. Thus, NGS should currently be viewed as a complementary approach rather than a replacement for established biochemical screening. Future prospective studies incorporating clinical outcomes, economic evaluations, and long-term follow-up, together with standardized quality control for integrated MS/MS–NGS workflows, are needed to clarify its appropriate role in population-based screening.

### 7.3. Continuity of Care, Family Support, and Ethics

Incomplete recall, diagnosis, and follow-up remain important challenges, particularly for conditions newly added to regional screening panels. These gaps limit the clinical benefits of early detection, a problem further compounded by variability in informed consent, genetic privacy concerns, and result interpretation in genomic screening. Limited public awareness, especially among rural and migrant populations, together with insufficient genetic counseling capacity, further restricts the translation of screening findings into effective clinical management.

Future efforts should focus on strengthening the screening-to-care pathway by establishing standardized informed consent frameworks for genomic screening, developing long-term follow-up cohorts to evaluate intervention effectiveness and clinical outcomes, and expanding genetic counseling capacity to support both panel expansion and genomic integration. These measures will be essential to ensure that advances in screening technologies are accompanied by appropriate clinical management, ethical governance, and family support.

## 8. Conclusions

China has developed a large-scale NBS system over the past four decades, with expanding disease panels and increasing integration of genomic technologies. However, this review highlights a persistent gap between screening expansion and the development of standardized diagnostic pathways, equitable financing mechanisms, and region-specific evidence. Future progress should therefore prioritize evidence-based expansion, quality assurance, and equitable access rather than coverage alone. These efforts will be essential for building a more sustainable and patient-centered NBS system.

## Figures and Tables

**Table 1:** Major legislative, regulatory, and policy milestones in NBS (1994–2023).

Year	Policy/Regulation	Main Contribution	Ref.
1994	Law of the People’s Republic of China on Maternal and Infant Health Care (revised in 2009 and 2017)	Proposed gradual implementation of NBS and legally established the right to screening	[19]
2001	Measures for Implementation of the Law of the People’s Republic of China on Maternal and Infant Care (revised in 2017, 2022, and 2023)	Formally incorporated NBS into maternal and infant health technical services	[20]
2002	China Action Plan for Improving Birth Population Quality, Reducing Birth Defects and Disabilities (2002–2010)	Integrated NBS into the three-tiered birth defect prevention system	[21]
2004	Technical Specifications for Newborn Disease Screening (revised in 2010)	First standardization of DBS collection, laboratory testing, hearing screening, follow-up, and PKU/CH diagnosis-treatment	[22,23]
2009	Management of Screening for Neonatal Diseases	Clarified responsibilities of health departments, NBS centers, and medical institutions; incorporated CH, PKU, and hearing loss into national program	[24]
2009	National Neonatal Disease Screening Work Plan	Set 2015 regional screening rate targets: NBS 95%/80%/60% (Easter/Central/Western); hearing screening 90%/60%/50% (Easter/Central/Western)	[25]
2009–2019	PKU Treatment Assistance Program funded by government	Phase 1 (2009): free phenylalanine-free formula for 500 disadvantaged children across 21 provinces (3 years); Phase 2 (2016–2019): added caregiver education, generating evidence for nationwide scale-up	[26,27]
2012–2016	Newborn Disease Screening Program in Poverty-Stricken Area, funded by government	Free PKU/CH screening piloted in Yunnan, Xinjiang, etc. (2012); hearing screening added (2013); expanded to 364 counties/21 provinces (2014); RMB 550 million invested, benefiting 4.38 million newborns (by Sept 2016)	[28,29,30]
2016	Healthy China 2030 Planning Outline	Emphasized expanding NBS coverage and neonatal critical treatment capacity	[31]
2018	The National Comprehensive Prevention and Treatment Program for Birth Defects	Gradual expansion of screened conditions and 2022 targets (98% IEM, 90% hearing, 80% treatment)	[32]
2023	Birth Defect Prevention and Treatment Capacity Enhancement Plan (2023–2027)	Expanded panels (target: 98% IEMs, 90% hearing by 2027); shifted focus to timeliness—diagnosis/treatment within 2 weeks (PKU and CH), 3 months diagnosis/6 months intervention (hearing); congenital heart disease screening nationwide by 2025, 80% screening/diagnosis/intervention by 2027	[33]

**Table 2:** Three-tier collaborative governance architecture and institutional responsibilities for NBS in China.

Governance Level	Primary Function	Leading Institution	Main Responsibilities
National	Policy and Planning	Department of Maternal and Child Health Services, National Health Commission	(1) Formulating national policies; (2) Defining technical standards and disease panels; (3) Coordinating resource allocation; (4) Developing information platforms; (5) Integrating data into national birth defects surveillance.
Provincial	Supervision and Implementation	Provincial Health Commissions	(1) Developing regional implementation plans; (2) Supervising the full NBS process (DBS collection, laboratory testing, case recall, diagnosis, treatment); (3) Personnel training; (4) Financial management; (5) Introducing new technologies.
Prefectural/County	Execution and Service	Maternal and Child Health Institutions	(1) Organizing and implementing screening; (2) Ensuring quality control and timely sample delivery; (3) Recalling and following up screen-positive infants; (4) Data reporting.

**Table 3:** EQA participation growth in China’s NBS laboratories.

Screening Category	Baseline Year	Baseline (No. of Labs)	2023 (No. of Labs)
Phenylalanine & TSH	1998	18	261
G6PD	2012	77	191
17-hydroxyprogesterone	2013	52	236
MS/MS (amino acids & acylcarnitine) *	2013	32	359

* Indicates an additional 76 laboratories participating in the EQA program for MS-based urine organic acid testing.

**Table 4:** Newborns screened and confirmed diagnoses of CH and PKU in the NBS program in China, 1985–2024.

Year	CH			PKU		
	Newborns Screened	Diagnosis Number	Incidence Rate	Newborns Screened	Diagnosis Number	Incidence Rate
1985	-	-	-	52,722	5	1/10,544
1986	-	-	-	69,506	2	1/34,753
1987	-	-	-	83,262	5	1/16,652
1988	-	-	-	67,535	5	1/13,507
1989	-	-	-	65,722	2	1/32,861
1990	-	-	-	49,033	3	1/16,344
1991	150,801	37	1/4076	50,113	4	1/12,528
1992	75,836	16	1/4740	117,904	6	1/19,651
1993	121,103	18	1/6728	121,106	10	1/12,111
1994	130,875	19	1/6888	126,141	4	1/31,535
1995	123,494	25	1/4940	121,227	24	1/5010
1996	173,707	31	1/5603	164,509	13	1/12,655
1997	292,583	65	1/4501	280,891	22	1/12,768
1998	400,653	137	1/2924	385,297	35	1/11,008
1999	477,282	189	1/2525	471,405	59	1/7990
2000	686,777	308	1/2229	686,075	55	1/12,474
2001	851,855	319	1/2670	852,605	76	1/11,218
2002	949,495	474	1/2003	950,960	95	1/10,010
2003	1,427,596	886	1/1611	1,367,541	155	1/8822
2004	1,911,349	998	1/1915	2,021,541	156	1/12,959
2005	2,511,814	1282	1/1959	2,632,419	213	1/12,358
2006	2,944,022	1701	1/1730	2,929,236	221	1/13,254
2007	5,603,451	2693	1/2081	5,289,471	468	1/11,302
2008	7,375,993	3471	1/2123	7,375,993	818	1/9009
2009	8,512,368	4172	1/2041	8,559,080	934	1/9091
2010	9,753,565	4519	1/2159	9,753,565	746	1/13,075
2011	11,144,495	5134	1/2171	11,144,495	778	1/14,325
2012	13,277,938	5004	1/2635	13,277,938	879	1/15,106
2013	14,499,407	5855	1/2476	14,499,407	923	1/15,709
2014	15,356,369	6222	1/2468	15,356,369	971	1/15,815
2015	15,090,978	6500	1/2322	15,090,978	979	1/15,415
2016	15,778,049	6515	1/2421	15,778,049	1,027	1/15,363
2017	17,509,788	8529	1/2052	17,509,788	1,266	1/13,830
2018	14,712,592	7768	1/1894	14,713,050	925	1/15,906
2019	14,113,980	7974	1/1770	14,090,430	930	1/15,151
2020	11,861,852	7556	1/1570	11,818,182	780	1/15,152
2021	10,393,895	7121	1/1460	10,323,529	702	1/14,706
2022	9,389,802	6892	1/1362	9,389,802	630	1/14,904
2023	8,601,000	6948	1/1238	8,601,000	630	1/13,652
2024	9,416,000	8849	1/1064	9,416,000	680	1/13,847
Total	225,620,764	118,227	1/1908	225,653,876	16,236	1/13,898

**Table 5:** Regional incidence of IEMs in China.

Geographic Region	Location	Population Screened	Incidence of IEMs	Ref.
Eastern	Shanghai	1,170,000	1/3000	[59]
	Zhejiang (2009–2021)	4,706,916	1/4535	[60]
	Jiangsu	245,194	1/2581	[61]
	Suzhou	—	1/3163 *	[11]
	Jining	514,234	1/1941	[62]
	Quanzhou	364,545	1/2804	[63]
Southern	Guangzhou	272,117	1/2451	[64]
Western	Xinjiang	107,741	1/1476	[65]
	Xi’an	146,152	1/1898	[66]
Central	Huaihua	206,977	1/3000	[67]
	Changsha	300,849	1/4237	[68]

* Excluding SCADD and 3-MCCD.

## Data Availability

The processed data presented in this study are available on reasonable request from the corresponding author.

## Abbreviations

The following abbreviations are used in this manuscript:

CAH	Congenital adrenal hyperplasia
CH	Congenital hypothyroidism
DBS	Dried blood spot
DMD	Duchenne muscular dystrophy
EQA/PT	External Quality Assessment/Proficiency Testing
G6PD	Glucose-6-phosphate dehydrogenase
IAEA	International Atomic Energy Agency
IEMs	Inborn errors of metabolism
IQC	Internal Quality Control
LSDs	Lysosomal storage diseases
MS/MS	Tandem mass spectrometry
NBS	Newborn screening
NBGS	Newborn genetic screening
NCCL	National Center for Clinical Laboratories
NGS	Next-generation sequencing
PKU	Phenylketonuria
PPV	Positive predictive value
QIS	Quality indicator system
QMIP	Quality management implementation program
SCID	Severe combined immunodeficiency
SMA	Spinal muscular atrophy
WGS	Whole-genome sequencing
X-ALD	X-linked adrenoleukodystrophy
XLA	X-linked Agammaglobulinemia

## References

[1] Jiang L.H., Yang R.L., Dong A., Wu B.Q., Zhao Z.Y. (2023). Progress of Newborn Screening in China. J. Zhejiang Univ. (Med. Sci.).

[2] Cao J., Pasquali M., Jones P.M. (2024). Newborn Screening: Current Practice and Our Journey over the Last 60 Years. J. Appl. Lab. Med..

[3] El-Hattab A.W., Almannai M., Sutton V.R. (2018). Newborn Screening: History, Current Status, and Future Directions. Pediatr. Clin. N. Am..

[4] Shao X., Steiner R., Peterson A.L. (2024). Newborn screening for lipid disorders. Curr. Opin. Lipidol..

[5] Alhusseini N., Almuhanna Y., Alabduljabbar L., Alamri S., Altayeb M., Askar G., Alsaadoun N., Ateq K., AlEissa M.M. (2024). International Newborn Screening: Where Are We in Saudi Arabia?. J. Epidemiol. Glob. Health.

[6] Kingsmore S.F., Smith L.D., Kunard C.M., Bainbridge M., Batalov S., Benson W., Blincow E., Caylor S., Chambers C., Del Angel G. (2022). A genome sequencing system for universal newborn screening, diagnosis, and precision medicine for severe genetic diseases. Am. J. Hum. Genet..

[7] Chien Y.-H., Hwu W.-L. (2022). The modern face of newborn screening. Pediatr. Neonatol..

[8] Bhattacharya K., Wotton T., Wiley V. (2014). The evolution of blood-spot newborn screening. Transl. Pediatr..

[9] Chace D.H. (2008). Mass spectrometry in newborn and metabolic screening: Historical perspective and future directions. J. Mass Spectrom..

[10] Millington D.S., Kodo N., Norwood D.L., Roe C.R. (1990). Tandem mass spectrometry: A new method for acylcarnitine profiling with potential for neonatal screening for inborn errors of metabolism. J. Inherit. Metab. Dis..

[11] Wang T., Ma J., Zhang Q., Gao A., Wang Q., Li H., Xiang J., Wang B. (2019). Expanded Newborn Screening for Inborn Errors of Metabolism by Tandem Mass Spectrometry in Suzhou, China: Disease Spectrum, Prevalence, Genetic Characteristics in a Chinese Population. Front. Genet..

[12] Stark Z., Scott R.H. (2023). Genomic newborn screening for rare diseases. Nat. Rev. Genet..

[13] Sheridan C. (2025). Baby genomics: Newborn sequencing starts to fulfill its promise. Nat. Biotechnol..

[14] Zhao Z.Y. (2024). Forty-three years of development of newborn screening in China: From Professor Chen Ruiguan. J. Clin. Pediatr..

[15] Zhao Z., Gu X. (2025). Newborn Screening.

[16] Padilla C.D., Therrell B.L. (2007). Newborn screening in the Asia Pacific region. J. Inherit. Metab. Dis..

[17] Therrell B.L., Padilla C.D., Abadingo M.E., Adhikari S.P., Aung T., Aye T.T., Dey S.K., Faizi M., Ganbaatar E., Giang T.T.H. (2024). Consolidated Newborn Bloodspot Screening Efforts in Developing Countries in the Asia Pacific-2024. Int. J. Neonatal Screen..

[18] Moslem F., Yasmeen S., Hasan M., Karim M.A., Nahar N., Ahmed A. (2003). Newborn screening: Experience of Bangladesh. Southeast Asian J. Trop. Med. Public Health.

[19] The State Council of the People’s Republic of China The Law of the People’s Republic of China on Maternal and Child Health Care. https://www.gov.cn/guoqing/2021-10/29/content_5647619.htm.

[20] The State Council of the People’s Republic of China Measures for the Implementation of the Law of the People’s Republic of China on Maternal and Child Health Care. https://www.nhc.gov.cn/fzs/c100048/202303/bc5e8bb6c90e4f9e9059b039736bd165.shtml.

[21] Ministry of Health of the People’s Republic of China, China Disabled Persons’ Federation (2002). China Action Plan for Improving Birth Population Quality, Reducing Birth Defects and Disabilities (2002–2010). Chin. J. Reprod. Health.

[22] (2005). Notice of the Ministry of Health on Issuing the Technical Specifications for Newborn Disease Screening.

[23] (2011). Notice of the Ministry of Health on Issuing the Technical Specifications for Newborn Disease Screening.

[24] (2009). Measures for the Management of Newborn Disease Screening.

[25] Ministry of Health of the People’s Republic of China (2010). National Plan for Newborn Disease Screening Work. Chin. J. Reprod. Health.

[26] Liu Y.D., Zhang Q., Zhang Y.J., Yu E.L., Zhu J. (2012). Preliminary analysis of the effect of free special milk powder subsidy for children with phenylketonuria. Matern. Child. Health Care China.

[27] Liao L.H., Zhu J., Yu E.L., Deng K., Li Q., Liu Z., Xiang L.C., Li X.H. (2022). Clinical implementation effects of the second phase of the special milk powder subsidy project for children with phenylketonuria. Chin. J. Obstet. Gynecol. Pediatr. (Electron. Ed.).

[28] National Health and Family Planning Commission, China Disabled Persons’ Federation (2013). Notice on Issuing the Program for Newborn Disease Screening Project in Poverty-Stricken Areas. https://www.nhc.gov.cn/fys/c100078/201311/6ed9ecf5086148d3afa36249118fa40d.shtml.

[29] National Health and Family Planning Commission, China Disabled Persons’ Federation (2014). Notice on Issuing the Program for Newborn Disease Screening Project in Poverty-Stricken Areas. https://www.nhc.gov.cn/fys/c100078/201412/25b50d641b2741dda8b52d87682b1d29.shtml.

[30] Department of Maternal and Child Health, National Health Commission of the People’s Republic of China Major Public Health Service Programs for Maternal and Child Health Progressing Smoothly. https://www.nhc.gov.cn/fys/c100077/201404/dcc2d80fbc804644a6ca44fe36a77549.shtml.

[31] State Council of China (2016). Healthy China 2030 Planning Outline.

[32] (2018). Notice on Issuing the National Comprehensive Prevention and Treatment Program for Birth Defects.

[33] (2023). Notice of the General Office of the National Health Commission on Issuing the Plan for Enhancing Birth Defect Prevention and Treatment Capacity (2023–2027).

[34] The Standing Committee of the Beijing Municipal People’s Congress Beijing Municipal Implementation Measures for the Law of the People’s Republic of China on Maternal and Child Health Care. https://www.beijing.gov.cn/zhengce/dfxfg/202103/t20210313_2306735.html.

[35] The Standing Committee of the Beijing Municipal People’s Congress Shanghai Municipal Maternal and Child Health Care Regulations. https://wsjkw.sh.gov.cn/fg2/20180525/0012-31553.html.

[36] The Standing Committee of the Beijing Municipal People’s Congress Sichuan Provincial Implementation Measures for the Law of the People’s Republic of China on Maternal and Child Health Care. https://www.scspc.gov.cn/flfgk/scfg/200206/t20020625_12530.html.

[37] Health Commission of Guangdong Province (2019). Administrative Measures for Newborn Disease Screening in Guangdong Province.

[38] Office of the Health Commission of Zhejiang Province Notice on the Issuance of Zhejiang Province Newborn Disease Screening Project Implementation Plan (2023 Edition). https://zjjcmspublic.oss-cn-hangzhou-zwynet-d01-a.internet.cloud.zj.gov.cn/jcms_files/jcms1/web2760/site/attach/0/0a213c2c7a814d7d982439aef31ffa39.pdf.

[39] (2023). Neonatal Inherited Metabolic Disease Screening Group; Birth Defect Prevention and Control Professional Committee; Chinese Preventive Medicine Association. Expert Consensus on Organizational Management and Dried Blood Spot Collection for Neonatal Inherited Metabolic Disease Screening. Chin. J. Neonatol..

[40] Department of Medical Administration, National Health Commission of the People’s Republic of China Notice of the Ministry of Health on Issuing the “Administrative Measures for Clinical Laboratories in Medical Institutions”. https://www.nhc.gov.cn/yzygj/c100068/200603/508cbe26295944c0a1ed2999b25ee375.shtml.

[41] China National Accreditation Service for Conformity Assessment Application Requirements of Accreditation Criteria for the Quality and Competence of Medical Laboratories. https://www.cnas.org.cn/rkgf/sysrk/rkyyzz/art/2024/art_9a46afffeaa144c8b2e065f691045aea.html.

[42] Zhong K., Wang W., He F., Wang Z. (2015). Neonatal screening external quality assessment in China, 2014. J. Med. Screen..

[43] Wang W., Zhang X.Y., Yuan S., He F.L., Zhong K., Wang Z.G. (2017). National external quality assessment of quality indicators for newborn inherited metabolic disease screening. Chin. J. Child. Health Care.

[44] (2023). Conformity Assessment—General Requirements for the Competence of Proficiency Testing Providers.

[45] China National Accreditation Service for Conformity Assessment (2024). CNAS-CL03:2024 Accreditation Criteria for Proficiency Testing Providers.

[46] (2022). Medical Laboratories-Requirements for Quality and Competence.

[47] Wang J.X., Du Y.X., Yu C.W., Wang J. (2023). Evaluation and optimization suggestions for the consensus on quality indicators for newborn inherited metabolic disease screening. Shanghai Med. J..

[48] Expert Group on Newborn Inherited Metabolic Disease Screening Laboratories, National Center for Clinical Laboratories (2017). Consensus on quality indicators for newborn inherited metabolic disease screening. Chin. J. Lab. Med..

[49] Jin L., He F., Zhang C. (2023). Quality management of the newborn screening network: The Chinese experience. Chin. Med. J..

[50] Zhao Z., Gu X. (2015). Newborn Genetic Metabolic Diseases Screening.

[51] Zhao Z., Zhou W., Liang D. (2022). Newborn Genetic Screening.

[52] Xiang L., Tao J., Deng K., Li X., Li Q., Yuan X., Liang J., Yu E., Wang M., Wang H. (2019). Phenylketonuria incidence in China between 2013 and 2017 based on data from the Chinese newborn screening information system: A descriptive study. BMJ Open.

[53] Yao Y.-N., Yuan X.-L., Zhu J., Xiang L.-C., Li Q., Deng K., Li X.-H., Liu H.-M. (2021). Geographic variations in the incidence of congenital hypothyroidism in China: A retrospective study based on 92 million newborns screened in 2013-2018. Chin. Med. J..

[54] Yao Y., Deng K., Zhu J., Xiang L., Yuan X., Li Q., Liu L., Xu W. (2023). Increased incidence of congenital hypothyroidism in China: An analysis of 119 million screened newborns. Eur. J. Pediatr..

[55] Liu Z., Yu C., Li Q., Cai R., Qu Y., Wang W., Wang J., Feng J., Zhu W., Ou M. (2019). Chinese newborn screening for the incidence of G6PD deficiency and variant of G6PD gene from 2013 to 2017. Hum. Mutat..

[56] Li Z., Huang L., Du C., Zhang C., Zhang M., Liang Y., Luo X. (2021). Analysis of the Screening Results for Congenital Adrenal Hyperplasia Involving 7.85 Million Newborns in China: A Systematic Review and Meta-Analysis. Front. Endocrinol..

[57] Shi X.T., Cai J., Wang Y.Y., Tu W.J., Wang W.P., Gong L.M., Wang D.W., Ye Y.T., Fang S.G., Jing P.W. (2013). Newborn Screening for Inborn Errors of Metabolism in Mainland China: 30 Years of Experience.

[58] Deng K., Zhu J., Yu E., Xiang L., Yuan X., Yao Y., Li X., Liu H. (2020). Incidence of inborn errors of metabolism detected by tandem mass spectrometry in China: A census of over seven million newborns between 2016 and 2017. J. Med. Screen..

[59] Hao L., Liang L., Gao X., Zhan X., Ji W., Chen T., Xu F., Qiu W., Zhang H., Gu X. (2023). Screening of 1.17 million newborns for inborn errors of metabolism using tandem mass spectrometry in Shanghai, China: A 19-year report. Mol. Genet. Metab..

[60] Chen C., Xu Y.H., Wu C.L., Xu Y.H., Mao H.Q., Yang R.L. (2022). Quality evaluation of tandem mass spectrometry screening for neonatalinherited metabolic diseases in Zhejiang Province from 2009 to 2021. Prev. Med..

[61] Men S., Liu S., Zheng Q., Yang S., Mao H., Wang Z., Gu Y., Tang X., Wang L. (2023). Incidence and genetic variants of inborn errors of metabolism identified through newborn screening: A 7-year study in eastern coastal areas of China. Mol. Genet. Genom. Med..

[62] Yang C., Zhou C., Xu P., Jin X., Liu W., Wang W., Huang C., Jiang M., Chen X. (2020). Newborn screening and diagnosis of inborn errors of metabolism: A 5-year study in an eastern Chinese population. Clin. Chim. Acta.

[63] Lin Y., Zheng Q., Zheng T., Zheng Z., Lin W., Fu Q. (2019). Expanded newborn screening for inherited metabolic disorders and genetic characteristics in a southern Chinese population. Clin. Chim. Acta.

[64] Tang C., Tan M., Xie T., Tang F., Liu S., Wei Q., Liu J., Huang Y. (2021). Screening for neonatal inherited metabolic disorders by tandem mass spectrometry in Guangzhou. J. Zhejiang Univ. (Med. Sci.).

[65] Zhang H., Tian H., Dai W., Zhang L., Guo G., Ding G. (2025). Expanded newborn screening for inborn errors of metabolism and genetic variants in Xinjiang, China. Front. Genet..

[66] Zhang R., Qiang R., Song C., Ma X., Zhang Y., Li F., Wang R., Yu W., Feng M., Yang L. (2021). Spectrum analysis of inborn errors of metabolism for expanded newborn screening in a northwestern Chinese population. Sci. Rep..

[67] Xiao G., Feng Z., Xu C., Huang X., Chen M., Zhao M., Li Y., Gao Y., Wu S., Shen Y. (2024). 206,977 newborn screening results reveal the ethnic differences in the spectrum of inborn errors of metabolism in Huaihua, China. Front. Genet..

[68] Li X., He J., He L., Zeng Y., Huang X., Luo Y., Li Y. (2022). Spectrum Analysis of Inherited Metabolic Disorders for Expanded Newborn Screening in a Central Chinese Population. Front. Genet..

[69] Wang X., Sun Y., Guan X.-W., Wang Y.-Y., Hong D.-Y., Zhang Z.-L., Li Y.-H., Yang P.-Y., Jiang T., Xu Z.-F. (2025). Effect of newborn genomic screening for lysosomal storage disorders: A cohort study in China. Genome Med..

[70] Li R., Tian L., Gao Q., Guo Y., Li G., Li Y., Sun M., Yan Y., Li Q., Nie W. (2022). Establishment of Cutoff Values for Newborn Screening of Six Lysosomal Storage Disorders by Tandem Mass Spectrometry. Front. Pediatr..

[71] Chang S., Zhan X., Liu Y., Song H., Gong Z., Han L., Maegawa G.H.B., Gu X., Zhang H. (2024). Newborn Screening for 6 Lysosomal Storage Disorders in China. JAMA Netw. Open.

[72] Jia C., Zhao D., Li Y., Gao Y., Zhang X., Li X., Lv S., Li R., Zhu X., Liu S. (2022). Newborn screening and genomic analysis of duchenne muscular dystrophy in Henan, China. Clin. Chim. Acta.

[73] Jia X., Jiang X., Huang Y. (2022). A pilot study of newborn screening for Duchenne muscular dystrophy in Guangzhou. Heliyon.

[74] Jing M., Wang Y., Jing X.-Y., Mao X.-M. (2024). Screening for Duchenne muscular dystrophy in newborns in the Ningxia region. Chin. J. Contemp. Pediatr..

[75] Ke Q., Zhao Z.-Y., Griggs R., Wiley V., Connolly A., Kwon J., Qi M., Sheehan D., Ciafaloni E., Howell R.R. (2017). Newborn screening for Duchenne muscular dystrophy in China: Follow-up diagnosis and subsequent treatment. World J. Pediatr..

[76] Chen C., Zhang C., Wu D.-W., Wang B.-Y., Xiao R., Huang X.-L., Yang X., Gao Z.-G., Yang R.-L. (2024). Comprehensive newborn screening for severe combined immunodeficiency, X-linked agammaglobulinemia, and spinal muscular atrophy: The Chinese experience. World J. Pediatr..

[77] Tang C., Tang F., Cai Y., Tan M., Liu S., Xie T., Jiang X., Huang Y. (2023). A pilot study of newborn screening for X-linked adrenoleukodystrophy based on liquid chromatography-tandem mass spectrometry method for detection of C26:0-lysophosphatidylcholine in dried blood spots: Results from 43,653 newborns in a southern Chinese population. Clin. Chim. Acta.

[78] Chiang S.-C., Chen P.-W., Hwu W.-L., Lee A.-J., Chen L.-C., Lee N.-C., Chiou L.-Y., Chien Y.-H. (2018). Performance of the Four-Plex Tandem Mass Spectrometry Lysosomal Storage Disease Newborn Screening Test: The Necessity of Adding a 2nd Tier Test for Pompe Disease. Int. J. Neonatal Screen..

[79] Burlina A.B., Polo G., Rubert L., Gueraldi D., Cazzorla C., Duro G., Salviati L., Burlina A.P. (2019). Implementation of Second-Tier Tests in Newborn Screening for Lysosomal Disorders in North Eastern Italy. Int. J. Neonatal Screen..

[80] Priestley J.R.C., Adang L.A., Williams S.D., Lichter-Konecki U., Menello C., Engelhardt N.M., DiPerna J.C., DiBoscio B., Ahrens-Nicklas R.C., Edmondson A.C. (2022). Newborn Screening for X-Linked Adrenoleukodystrophy: Review of Data and Outcomes in Pennsylvania. Int. J. Neonatal Screen..

[81] Chen H.-A., Hsu R.-H., Chen P.-W., Lee N.-C., Chiu P.-C., Hwu W.-L., Chien Y.-H. (2022). High incidence of null variants identified from newborn screening of X-linked adrenoleukodystrophy in Taiwan. Mol. Genet. Metab. Rep..

[82] Luo X., Sun Y., Xu F., Guo J., Li L., Lin Z., Ye J., Gu X., Yu Y. (2020). A pilot study of expanded newborn screening for 573 genes related to severe inherited disorders in China: Results from 1,127 newborns. Ann. Transl. Med..

[83] Tang C., Li L., Chen T., Li Y., Zhu B., Zhang Y., Yin Y., Liu X., Huang C., Miao J. (2024). Newborn Screening for Inborn Errors of Metabolism by Next-Generation Sequencing Combined with Tandem Mass Spectrometry. Int. J. Neonatal Screen..

[84] Chen T., Fan C., Huang Y., Feng J., Zhang Y., Miao J., Wang X., Li Y., Huang C., Jin W. (2023). Genomic Sequencing as a First-Tier Screening Test and Outcomes of Newborn Screening. JAMA Netw. Open.

[85] Yang R.-L., Qian G.-L., Wu D.-W., Miao J.-K., Yang X., Wu B.-Q., Yan Y.-Q., Li H.-B., Mao X.-M., He J. (2023). A multicenter prospective study of next-generation sequencing-based newborn screening for monogenic genetic diseases in China. World J. Pediatr..

[86] Hao C., Guo R., Hu X., Qi Z., Guo Q., Liu X., Liu Y., Sun Y., Zhang X., Jin F. (2021). Newborn screening with targeted sequencing: A multicenter investigation and a pilot clinical study in China. J. Genet. Genom..

[87] Wang X., Sun Y., Guan X.-W., Wang Y.-Y., Hong D.-Y., Zhang Z.-L., Li Y.-H., Yang P.-Y., Jiang T., Xu Z.-F. (2023). Newborn genetic screening is highly effective for high-risk infants: A single-centre study in China. J. Glob. Health.

[88] Kingsmore S.F., Wright M., Smith L.D., Liang Y., Mowrey W.R., Protopsaltis L., Bainbridge M., Baker M., Batalov S., Blincow E. (2024). Prequalification of genome-based newborn screening for severe childhood genetic diseases through federated training based on purifying hyperselection. Am. J. Hum. Genet..

[89] Kingsmore S.F., Wright M., Olsen L., Schultz B., Protopsaltis L., Averbuj D., Blincow E., Carroll J., Caylor S., Defay T. (2024). Ge-nome-based newborn screening for severe childhood genetic diseases has high positive predictive value and sensitivity in a NICU pilot trial. Am. J. Hum. Genet..

[90] Ziegler A., Koval-Burt C., Kay D.M., Suchy S.F., Begtrup A., Langley K.G., Hernan R., Amendola L.M., Boyd B.M., Bradley J. (2024). Expanded Newborn Screening Using Genome Sequencing for Early Actionable Conditions. JAMA.

[91] Boemer F., Hovhannesyan K., Piazzon F., Minner F., Mni M., Jacquemin V., Mashhadizadeh D., Benmhammed N., Bours V., Jacquinet A. (2025). Population-based, first-tier genomic newborn screening in the maternity ward. Nat. Med..

[92] Huang S.Z., Song F., Writing Group for Clinical Practice Guidelines for Genetic Diseases, Chinese Society of Medical Genetics (2020). Clinical practice guidelines for phenylketonuria. Chin. J. Med. Genet..

[93] Chinese Fabry Disease Expert Collaborative Group (2021). Expert consensus on diagnosis and treatment of Fabry disease in China (2021 edition). Chin. J. Intern. Med..

[94] NeuroScience Professional Committee, Chinese Research Hospital Association, Special Fund Expert Group for Neuromuscular Disease Prevention and Control, China Birth Defects Intervention and Assistance Foundation (2023). Expert consensus on newborn screening for spinal muscular atrophy (2023 edition). Natl. Med. J. China.

[95] Working Group on Guidelines for Diagnosis and Treatment of Congenital Hypothyroidism, Chinese Society of Endocrinology, Chinese Pediatric Society (2025). Guidelines for diagnosis and treatment of congenital hypothyroidism. Chin. J. Endocrinol. Metab..

[96] Zhuang D.Y. (2020). Expert consensus on biological sample management for newborn disease screening. J. Clin. Lab. Sci..

[97] Tong F., Wang J., Xiao R., Wu B.-B., Zou C.-C., Wu D.-W., Wang H., Zou H., Han L.-S., Yang L. (2022). Application of next generation sequencing in the screening of monogenic diseases in China, 2021: A consensus among Chinese newborn screening experts. World J. Pediatr..

[98] Neonatal Inherited Metabolic Disease Screening Group, Birth Defect Prevention and Control Professional Committee, Chinese Preventive Medicine Association, National Committee for External Quality Assessment of Neonatal Inherited Metabolic Disease Screening, National Center for Clinical Laboratories (2023). Expert consensus on laboratory testing technical guidelines for neonatal inherited metabolic disease screening. Chin. J. Neonatol..

[99] Huang X.-W., Zhang T., Hu Z.-Z., Wang Z.-G., Luo X.-P., Yang Y.-L., Han L.-S., Gu X.-F., Xiao G.-R., Zhu B.-S. (2025). Expert consensus on the combined screening of genes and biomarkers for neonatal diseases. World J. Pediatr..

[100] Ou M.C., Jiang J.H., National Committee for External Quality Assessment of Neonatal Inherited Metabolic Disease Screening, National Center for Clinical Laboratories (2020). Expert consensus on follow-up of newborn screening for neonatal genetic and metabolic diseases. Chin. J. Med. Genet..

[101] (2023). Neonatal Inherited Metabolic Disease Screening Group; Birth Defect Prevention and Control Professional Committee; Chinese Preventive Medicine Association. Expert consensus on diagnosis and treatment of inherited metabolic diseases by newborn screening. Chin. J. Neonatol..

[102] Wang X., Guan X.-W., Wang Y.-Y., Zhang Z.-L., Li Y.-H., Yang P.-Y., Sun Y., Jiang T. (2022). Current attitudes and preconceptions on newborn genetic screening in the Chinese reproductive-aged population. Orphanet J. Rare Dis..

[103] The People’s Government of Kunshan City Notice on the Issuance of the Implementation Plan for the Free Screening Project of 29 Genetic Metabolic Diseases for Newborns with Household Registration in Kunshan City. https://www.ks.gov.cn/kss/c113206puf/201604/f1543901862145b8ac3e0bf5cc957041.shtml.

[104] The People’s Government of Kunshan City Fucheng Has Launched a Public Welfare Project to Subsidize the Screening of 48 Genetic Metabolic Diseases for Newborns. https://www.my.gov.cn/mysrmzf/c100089/202506/0cf335f10a6845dca7f91d6ae10e660d.shtml.

[105] The People’s Government of Sichuan Province (2025). The Free Genetic Metabolic Disease Screening for Newborns in Chengdu Has Benefited over 100,000 People This Year.

[106] Xiangtan Municipal Finance Bureau, Xiangtan Municipal Health Commission Notice on Allocating Funds for the 2024 Key Livelihood Project of Free Neonatal Disease Screening and Diagnosis by the Provincial Government. https://cz.xiangtan.gov.cn/2419/25302/content_1334430.html.

[107] Gongqingcheng Municipal Health Commission Gongqingcheng Municipal Health Commission Has Compiled the 2024 Departmental Budget. https://www.gongqing.gov.cn/zwgk/wugongkai/glgk/03/02/24/202402/t20240223_6415580.html.

[108] Mei L., Song P., Kokudo N., Xu L., Tang W. (2014). Current situation and prospects of newborn screening and treatment for Phenylketonuria in China—Compared with the current situation in the United States, UK and Japan. Intractable Rare Dis. Res..

[109] National Healthcare Security Administration What Are the Benefits for Newborns When They Enroll in Insurance?. https://www.nhsa.gov.cn/art/2025/7/16/art_14_17289.html.

[110] China Birth Defects Intervention Assistance Foundation Central Special Lottery Public Welfare Fund Project. https://www.csqx.org.cn/list.aspx?id=580303611982.

